# Pathogen Recognition Receptor Signaling Accelerates Phosphorylation-Dependent Degradation of IFNAR1

**DOI:** 10.1371/journal.ppat.1002065

**Published:** 2011-06-09

**Authors:** Juan Qian, Hui Zheng, Wei-Chun HuangFu, Jianghuai Liu, Christopher J. Carbone, N. Adrian Leu, Darren P. Baker, Serge Y. Fuchs

**Affiliations:** 1 Department of Animal Biology, School of Veterinary Medicine, University of Pennsylvania, Philadelphia, Pennsylvania, United States of America; 2 BiogenIdec, Cambridge, Massachusetts, United States of America; Washington University School of Medicine, United States of America

## Abstract

An ability to sense pathogens by a number of specialized cell types including the dendritic cells plays a central role in host's defenses. Activation of these cells through the stimulation of the pathogen-recognition receptors induces the production of a number of cytokines including Type I interferons (IFNs) that mediate the diverse mechanisms of innate immunity. Type I IFNs interact with the Type I IFN receptor, composed of IFNAR1 and IFNAR2 chains, to mount the host defense responses. However, at the same time, Type I IFNs elicit potent anti-proliferative and pro-apoptotic effects that could be detrimental for IFN-producing cells. Here, we report that the activation of p38 kinase in response to pathogen-recognition receptors stimulation results in a series of phosphorylation events within the IFNAR1 chain of the Type I IFN receptor. This phosphorylation promotes IFNAR1 ubiquitination and accelerates the proteolytic turnover of this receptor leading to an attenuation of Type I IFN signaling and the protection of activated dendritic cells from the cytotoxic effects of autocrine or paracrine Type I IFN. In this paper we discuss a potential role of this mechanism in regulating the processes of innate immunity.

## Author Summary

We have previously observed and reported that the activation of unfolded protein responses during infection with some viruses such as vesicular stomatitis virus and hepatitis C virus compromises the cellular responses to the cytokines that belong to family of Type I interferons (IFNα/β). These effects apparently rely on the activation of a specific protein kinase termed PERK that transduces signals from unfolded proteins to accelerate phosphorylation-dependent degradation of the IFNα/β receptor. Here we found that the same pathway can be PERK-independent and can be stimulated by signaling induced in the specialized cells (e.g. dendritic cells that produce IFNα/β), which recognize pathogens (e.g. viruses). This work delineates a mechanism by which the degradation of IFNα/β receptor is accelerated. Our studies also highlight the importance of this mechanism for limiting the magnitude and the duration of IFNα/β signaling and maintaining the survival of IFN-producing dendritic cells.

## Introduction

Cytokines that belong to the family of Type I interferons (IFNs, including IFNα/β), play an important role in innate immunity [1], [2], [3], [4]. These cytokines are produced by various cell types including dendritic cells (DCs), which are equipped with a diverse set of specialized pathogen recognition receptors (PRR) that recognize a variety of pathogens. Activation of PRR in DCs is known to induce the production of numerous cytokines including inflammatory cytokines and IFNα/β. The latter cytokines modulate the subsequent antigen-specific adaptive immune responses and mount the overall host defenses against infection and injury (reviewed in [5], [6], [7]).

Type I IFN mediates its effects through the stimulation of the Type I IFN receptor (which consists of IFNAR1 and IFNAR2 chains) and the subsequent activation of the Janus kinases TYK2 and JAK1, phosphorylation of STAT1/2 proteins, and ensuing trans-activation of a plethora of interferon-stimulated genes. The products of these genes directly suppress the spread of some pathogens (e.g., viruses) and cooperate with inflammatory cytokines in DCs to promote antigen presentation linking innate and adaptive immunity (reviewed in [8], [9], [10]). Some of these genes also elicit pronounced anti-proliferative and pro-apoptotic effects [10]. Conversely, cells that produce IFNα/β have to survive in this environment and have to be protected from autocrine/paracrine IFNα/β [11].

Accordingly, Type I IFNs play a dynamic role in homeostasis and in the function of DCs [12]. Whereas IFNα/β contribute to the maturation and activation of DCs [13], [14], [15], these cytokines are also known to decrease the viability of IFNα/β-producing DCs [4], [12], [16]. Additional mechanisms of negative regulation are expected to prevent a hypothetical scenario where IFNα/β-stimulated maturation of DCs and subsequent production of more of IFNα/β by these DCs spirals out of control and leads to a hyperactivation of pathways induced by Type I IFN. Such hyperactivation of the IFNα/β pathways might be detrimental, not only to a population of specific IFN-producing cells, but also to the entire host because it leads to autoimmune disorders. A key role of IFNα/β in pathogenesis of such disorders, including psoriases, systemic lupus erythematosus and Type 1 diabetes mellitus, has been thoroughly documented [17], [18], [19], [20]. Therefore, developing an understanding of the mechanisms, by which IFNα/β responses are kept in check, is of medical importance.

All responses to IFNα/β require the presence of the Type I IFN receptor at the cell surface [21]. Upon interacting with the ligands, this receptor not only conducts the forward signaling through the JAK-STAT pathway but also undergoes rapid downregulation [22], [23]. This downregulation is driven by the phosphorylation-dependent ubiquitination, endocytosis, and degradation of the IFNAR1 chain [24], [25]. Ubiquitination of IFNAR1 is facilitated by the SCF^βTrcp^ E3 ubiquitin ligase, which is recruited to the receptor upon phosphorylation on serine residues (S535 in humans, S526 in mice) within IFNAR1's degron [25], [26]. Such phosphorylation can be induced by a ligand in a TYK2 activity-dependent manner [27] through the activation of protein kinase D2 [28]. This ligand-inducible pathway limits the extent of IFNα/β signaling in the cells that have already been exposed to these cytokines.

We have recently discovered an alternative (TYK2 activity-independent) signaling pathway that leads to S535 phosphorylation and to the subsequent ubiquitination and downregulation of IFNAR1 in the absence of a ligand. As a result, IFNAR1 is being removed from the surface of those cells, which have not been yet exposed to IFNα/β [29]. Accordingly, this signaling does not require the ligand and hence can impair the ability of a naïve cell to respond to its future encounters with IFNα/β. Such a pathway can be further stimulated by the inducers of the unfolded protein response (UPR). UPR is a complex of signaling pathways that are essential for protein quality control in cells [30], [31] and can be induced by various stress stimuli such as thapsigargin (TG) as well as by viral infection [32]. Inducers of UPR (including RNA-containing viruses such as vesicular stomatitis virus, VSV, and hepatitis C virus, HCV) were shown to promote IFNAR1 degradation via a pathway that required the activation of a PKR-like ER kinase (PERK, [33]). Subsequent delineation of the ligand-independent pathway revealed an important role for casein kinase 1α (CK1α) in the direct phosphorylation of S535/S526 within the IFNAR1 degron [34]. The ability of constitutively active CK1α to phosphorylate the IFNAR1 degron was augmented in cells treated with UPR inducers or infected with VSV via priming phosphorylation of another serine – S532 (S523 in mouse IFNAR1). The latter phosphorylation was mediated by a yet to be identified kinase that functioned downstream of PERK [35].

Here we report that the DNA-containing herpes simplex virus (HSV) also induced the ligand/TYK2-independent phosphorylation and downregulation of IFNAR1. Remarkably, these effects required neither viral protein synthesis nor PERK activity but relied on the activation of the PRR signaling pathways that center around the activation of the p38 protein kinase. This kinase was required for the phosphorylation of the IFNAR1 priming site leading to an ensuing phosphorylation of the IFNAR1 degron on Ser535/526, acceleration of IFNAR1 degradation, and attenuation of IFNα/β signaling. These events appear to be important for the protection of DCs from autocrine/paracrine Type I IFN.

## Results

### Herpes Simplex Virus downregulates IFNAR1 via the ligand independent pathway

**Figure 1:**
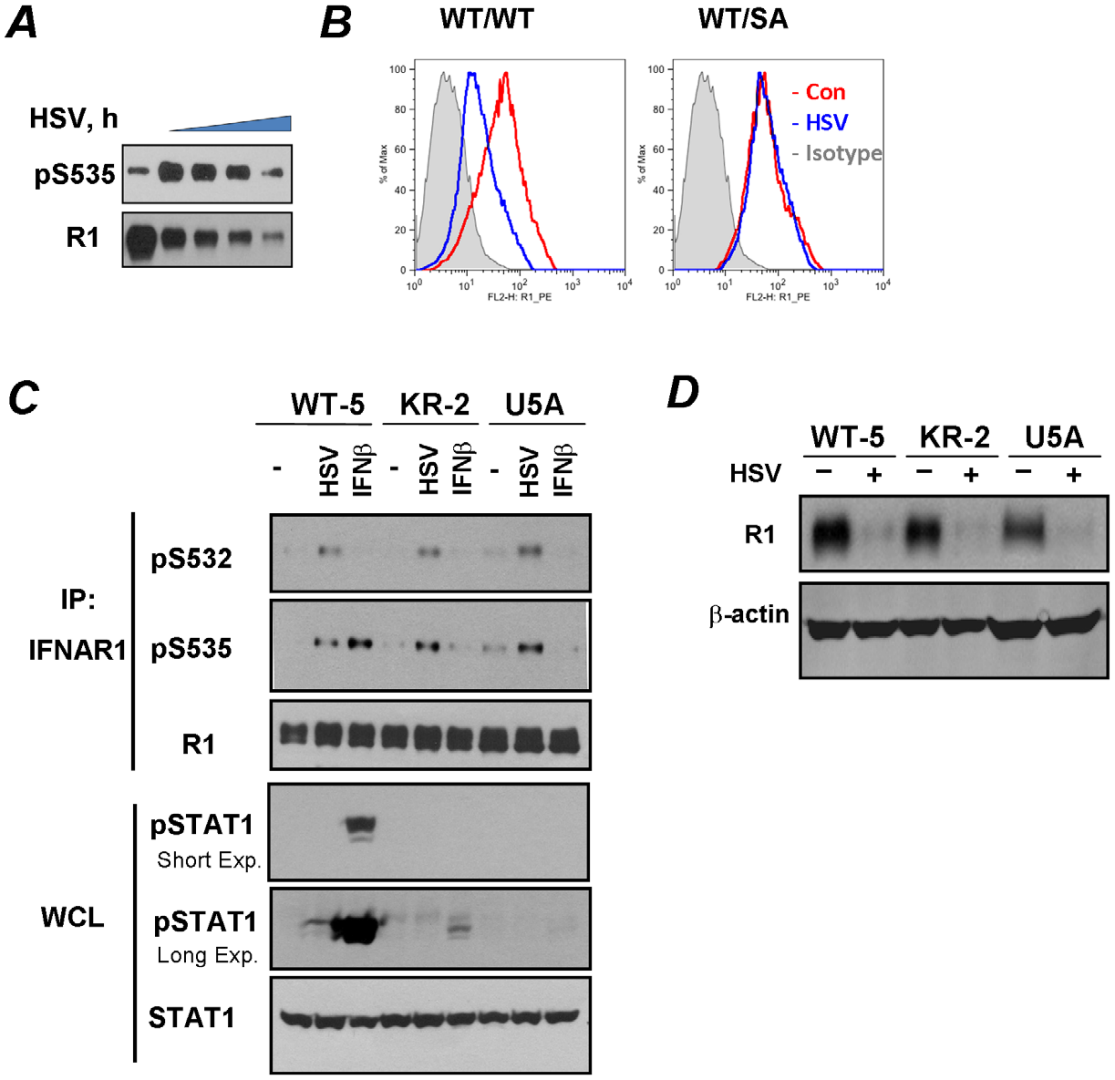
HSV induces ligand/TYK2-independent phosphorylation and downregulation of IFNAR1. **(A)** Human KR-2 cells were infected with HSV (MOI 0.1) and harvested at 0, 24, 26, 28, and 30 h post-infection. Cell lysates were immunoprecipitated (IP) using the anti-IFNAR1 (“R1”) antibody. The phosphorylation of IFNAR1 at Ser535 and total levels of IFNAR1 were analyzed by immunoblotting (IB) using the indicated antibodies. **(B)** MEF (from wild type or S526A mice) were uninfected (red line) or infected (blue line) with HSV (MOI 0.1 for 32 h). Cells were subsequently incubated with the anti-mouse IFNAR1 antibody, biotin-conjugated goat-anti-mouse IgG and PE-streptavidine and then analyzed by FACS (BD Caliber). Gray area depicts a control reaction with an isotype antibody. **(C)** Human WT-5, KR-2 or U5A fibrosarcoma cells were either infected with HSV (MOI 0.1 for 22 h) or treated with human IFNβ (1000 IU/ml for 30 min). The analysis of IFNAR1 levels and phosphorylation in IFNAR1 immunoprecipitates was carried out using the indicated antibodies. The analyses of STAT1 phosphorylation and levels in whole cell lysates (WCL) are also shown. Exp., exposure. **(D)** Human WT-5, KR-2 or U5A fibrosarcoma cells were left intact or infected with HSV (MOI 0.1 for 30 h). Analyses of IFNAR1 levels in IFNAR1 immunoprecipitates and β-actin (as a loading control) in the supernatants of the immunoprecipitation reactions were carried out using indicated antibodies.

RNA-containing viruses (HCV and VSV) can downregulate IFNAR1 in human KR-2 cells that harbor a catalytically inactive TYK2 and that are deficient in IFNα-stimulated Ser535 phosphorylation of IFNAR1 and the degradation of this receptor chain [27], [33]. We sought to investigate whether a DNA-containing virus such as the herpes simplex virus (HSV) is also capable of such activity. We observed that the infection of KR-2 cells with HSV stimulates degron phosphorylation of endogenous human IFNAR1 and robustly downregulates the levels of this receptor (Figure 1A). A marked decrease in cell surface levels of murine IFNAR1 in response to HSV infection was also seen in mouse embryo fibroblasts (MEFs, Figure 1B).

To determine whether downregulation of IFNAR1 by HSV depends on IFNAR1 degron phosphorylation, we have generated a knock-in mouse that expresses a phosphorylation-deficient IFNAR1 mutant. To this end, mouse ES cells, in which one wild type *Ifnar1* allele was replaced with a mutant allele that lacks Ser526 (a serine residue homologous to Ser535 within human IFNAR1; cells were described in [33]) were rid of the Neo cassette. They were then used to generate knock-in mice that express the IFNAR1^S526A^ mutant (“SA”, Figure S1). Importantly, MEFs derived from these mice were noticeably resistant to an HSV-induced downregulation of IFNAR1 levels on the cell surface (Figure 1B). This result suggests that HSV downregulates IFNAR1 in a degron phosphorylation-dependent manner.

Given that HSV has been known to induce the production of both IFNα and IFNβ [36], we next sought to delineate the mechanisms by which HSV infection stimulates the phosphorylation of IFNAR1 degron. We chose to analyze endogenous IFNAR1 in human fibrosarcoma-derived cell lines harvested at the earlier time points (20–22 hr) of HSV infection at low MOI (0.1). Under these conditions, the total levels of IFNAR1 were not yet dramatically downregulated, enabling a better detection of Ser535 phosphorylation. HSV infection of WT-5 cells that express wild type TYK2 [37], [38] induced a robust S535 phosphorylation of IFNAR1 (Figure 1C). Comparable levels of IFNAR1 degron phosphorylation were seen in the isogenic KR-2, which express kinase-dead TYK2 and are responsive to IFNβ [38] but not to IFNα [27], [37] suggesting that the activity of TYK2 is not required for the effects of the virus. On the contrary, the phosphorylation of IFNAR1 Ser535 in response to treatment with recombinant IFNβ was much less evident in KR-2 cells that displayed only a rather modest STAT1 phosphorylation in response to this cytokine (Figure 1C). Furthermore, in either WT-5 or KR-2 cells, infection with HSV hardly induced any STAT1 phosphorylation indicating that an increase in IFNAR1 degron phosphorylation may not rely on HSV-induced production of Type I IFN. Finally, in the isogenic fibrosarcoma U5A, cells that lack the IFNAR2 chain of the Type I IFN receptor and are insensitive to any Type I IFN [39], infection with HSV robustly induced Ser535 phosphorylation of IFNAR1 (Figure 1C). These results suggest that the effects of HSV on phosphorylation of IFNAR1 are ligand-independent.

These isogenic WT-5, KR-2 and U5A cell lines were infected with HSV for longer periods of time to determine the role of TYK2 kinase activity and endogenous Type I IFN in downregulation. A comparable decrease of IFNAR1 levels in response to infection was seen in WT-5 and KR-2 cells. Furthermore, HSV robustly decreased levels of IFNAR1 in U5A cells (Figure 1D). These results indicate that neither production of the endogenous ligands nor catalytic activities of TYK2 are required for IFNAR1 downregulation in cells infected with HSV.

**Figure 2:**
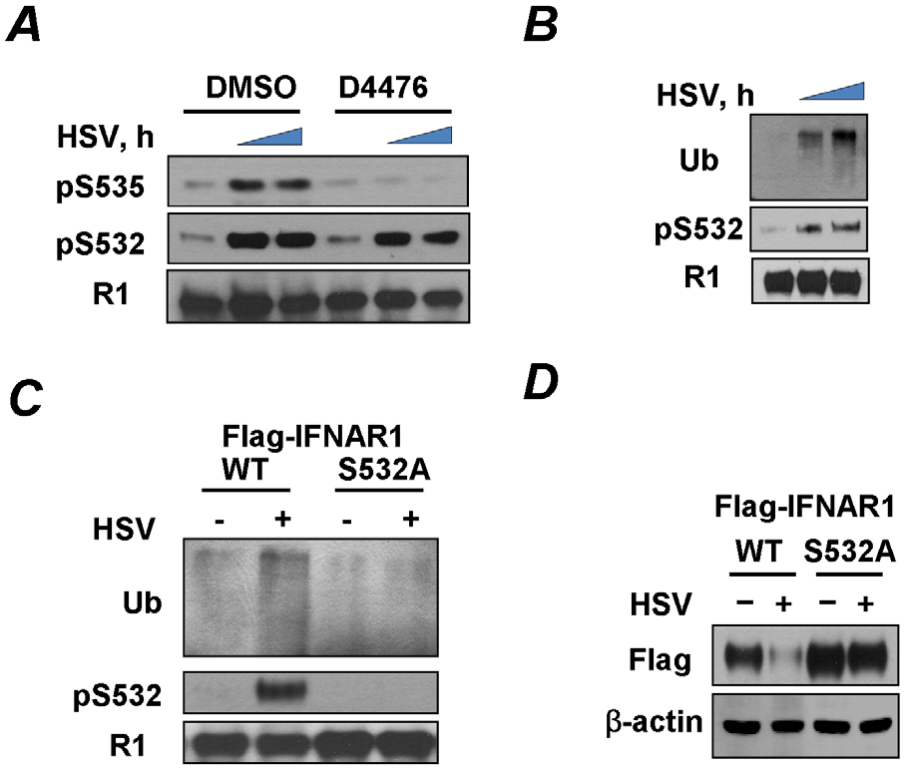
HSV induces priming phosphorylation-dependent ubiquitination and downregulation of IFNAR1. **(A)** KR-2 cells were infected with HSV (MOI 0.1) and were treated with either DMSO (control) or CK1 inhibitor D4476 (12.5 µM for 1 h) before harvesting at 0, 21, or 23 h post-infection. Cell lysates were immunoprecipitated with the IFNAR1 antibody and analyzed by IB with the indicated antibodies. **(B)** KR-2 cells were infected with HSV (MOI 0.1) and were harvested at 22 or 24 h post-infection. Cell lysates were analyzed by IFNAR1 IP followed by IB using antibodies against ubiquitin, phospho-S532, and IFNAR1. **(C)** KR-2 cells expressing Flag-IFNAR1 (wild type or S532A mutant) were infected with HSV (MOI 0.1) and harvested 24 h post-infection. The phosphorylation and ubiquitination of Flag-IFNAR1 were analyzed by IP using anti-Flag antibodies followed by an IB analysis as described in panel D. **(D)** KR-2 cells expressing the indicated Flag-IFNAR1 (wild type or S532A mutant) were infected with HSV (MOI 0.1) and harvested 30 h post-infection. Levels of exogenous IFNAR1 and β-actin were analyzed by IB using the indicated antibodies.

Given the latter results we sought to determine whether, similarly to VSV, the effects of HSV infection on IFNAR1 might be mediated by the constitutively active kinase CK1α [34] whose ability to phosphorylate IFNAR1 degron is augmented by a priming phosphorylation of IFNAR1 on S532 [35]. Phosphorylation of the degron (S535) in HSV-infected KR-2 cells was indeed attenuated by treating the cells with a CK1 inhibitor (Figure 2A). Furthermore, HSV infection induced the priming phosphorylation of IFNAR1 on S532 (Figures 1C and 2A–C) and IFNAR1 ubiquitination (Figures 2B–C). The IFNAR1^S532A^ mutant lacking the priming site was noticeably more resistant to the HSV-stimulated ubiquitination and downregulation than the wild type receptor (Figures 2C–D). Together, these results suggest that, similar to VSV, HSV stimulates the ligand/TYK2-independent pathway of IFNAR1 phosphorylation-dependent ubiquitination and downregulation that requires the priming phosphorylation.

### PERK is not required for phosphorylation and downregulation of IFNAR1 in response to HSV

**Figure 3:**
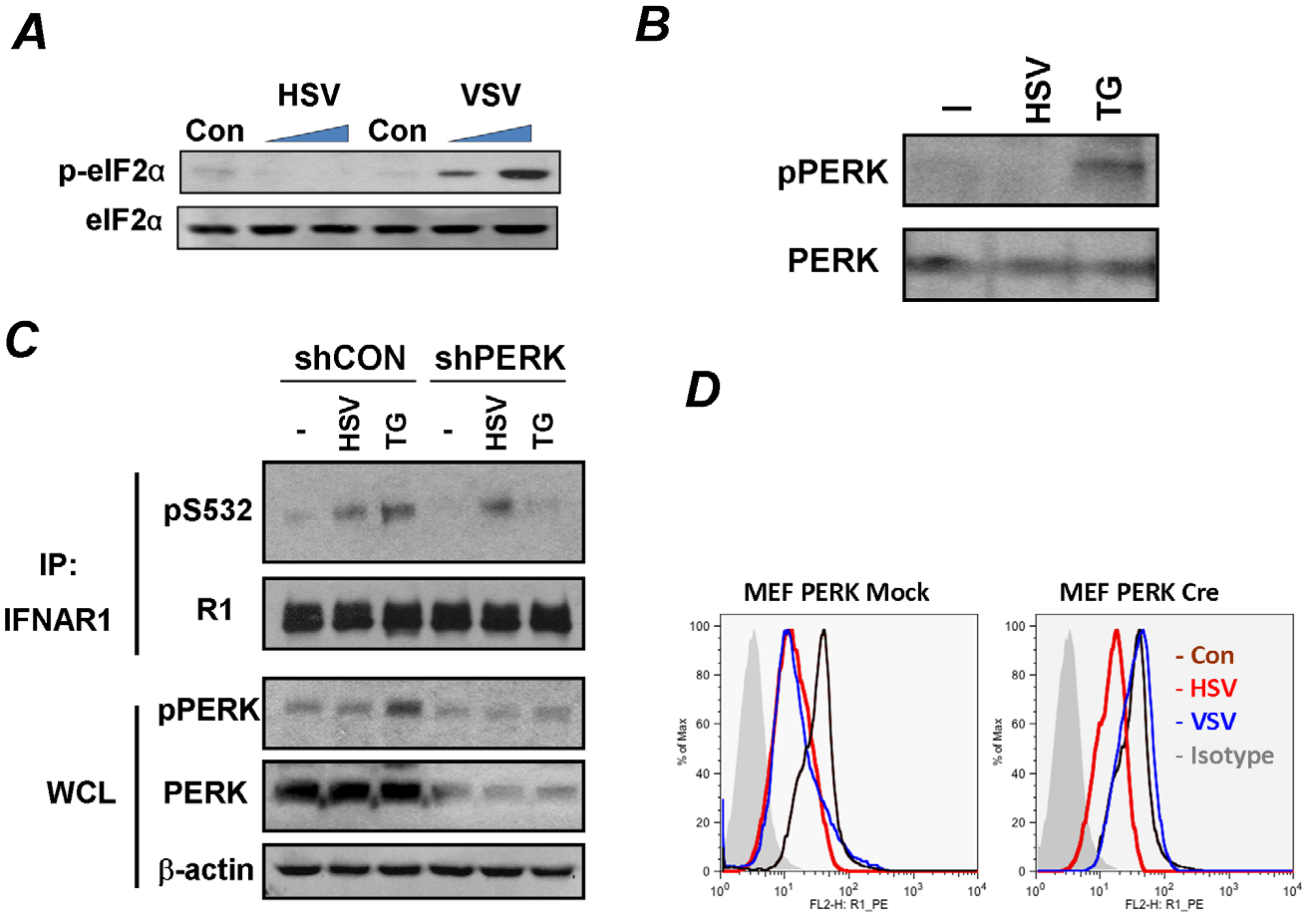
Phosphorylation-dependent downregulation of IFNAR1 by HSV occurs in a PERK-independent manner. **(A)** KR-2 cells were uninfected or infected with VSV (MOI 0.1 for 12 and 14 h) or HSV (MOI 0.1 for 14 and 30 h). Cell lysates were analyzed by IB using the indicated antibodies.**(B)** KR-2 cells were treated with thapsigargin (TG, 1 µM for 30 min) or infected with HSV (MOI 0.1 for 22 h). Phosphorylation and total levels of PERK were analyzed using the indicated antibodies. **(C)** KR-2 cells were transduced with lentiviral vectors that express shRNA against PERK or against GFP (shCON, used as a control) and treated or infected as described in panel B. The phosphorylation and levels of IFNAR1 in immunoprecipitates and phosphorylation and levels of PERK and β-actin in whole cell lysates (WCL) were analyzed using the indicated antibodies. **(D)** MEFs from PERK^fl/fl^ mice were transduced with an empty lentivirus (Mock) or lentiviral vector for the expression of Cre recombinase. Cells were then left untreated (magenta line) or infected with VSV (MOI 0.1 for 14 h, blue line) or HSV (MOI 0.1 for 30 h, red line). Cell surface IFNAR1 levels were analyzed by FACS.

The experimental settings of all these experiments included infection at low doses (MOI 0.1) that did not induce changes in the status of IFNAR1 (data not shown) until later periods of the infection (24–30 h post infection) which were chosen for the maximal expression of viral proteins to induce UPR. Under these conditions, HCV and VSV required PERK activity to promote IFNAR1 phosphorylation and degradation [33]. Although KR-2 cells display a noticeable increase in the phosphorylation of translational regulator eIF2α (a major PERK substrate) in response to thapsigargin or VSV [33], [35], stimulation of this signaling event by HSV infection was modest at best (Figure 2A). These results are consistent with previous reports that attributed low levels of eIF2α phosphorylation either to the stimulation of eIF2α de-phosphorylation by the HSV protein γ_1_34.5 under conditions where PERK is activated [40] or to the suppression of PERK activation by the viral glycoprotein B [41]. Under the conditions used in our experiments in KR-2 cells to influence the phosphorylation of IFNAR1 degron, an induction of PERK phosphorylation (indicative of its activation) was observed in response to thapsigargin but not to infection with HSV (Figures 3B–C). Both thapsigargin and HSV infection stimulated the priming phosphorylation of IFNAR1 on Ser532 in KR-2 cells. Whereas the knockdown of PERK noticeably attenuated the effects of thapsigargin, priming phosphorylation of IFNAR1 stimulated by, HSV infection was impervious to the modulations of PERK expression (Figure 3C). This result suggests that PERK is dispensable for HSV-stimulated IFNAR1 phosphorylation in human cells.

We further tested the requirement of PERK by using MEFs from mice harboring a conditional knockout allele of *PERK* (PERK^fl/fl^) where *PERK* is acutely excised upon transduction with a retrovirus encoding the Cre recombinase [42]. Consistent with a previous report [33], the acute deletion of PERK prevented the downregulation of IFNAR1 upon VSV infection. However, the downregulation of IFNAR1 stimulated by HSV was not affected by the status of PERK (Figure 3D). These data suggest that HSV is capable of stimulating the ligand-independent pathway in a manner that differs from viral protein synthesis-induced UPR and activation of PERK described for HCV and VSV.

**Figure 4:**
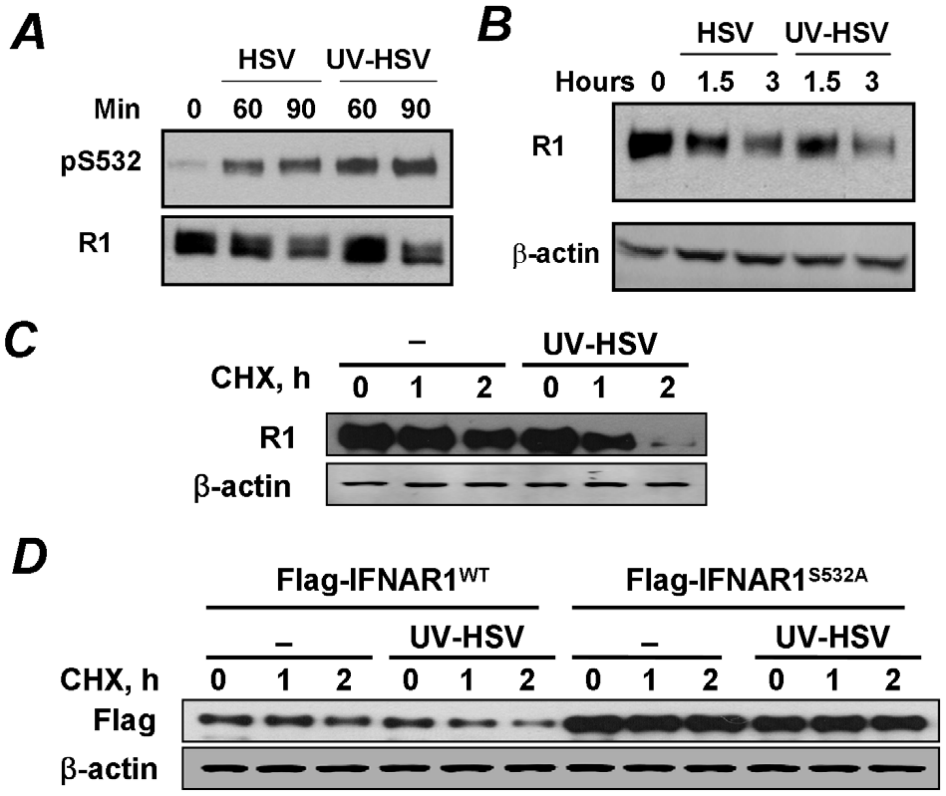
Phosphorylation, downregulation and degradation of IFNAR1 can be stimulated by inactivated HSV. **(A)** The stock of HSV was inactivated or not with UV and used to treat KR-2 cells at MOI 5.0 for indicated time points. Cell lysates were analyzed by IP using the anti-IFNAR1 antibody and IB using indicated antibodies. **(B)** KR-2 cells were treated with either UV- or sham-treated HSV as shown in panel A. The levels of IFNAR1 and β-actin were assessed as in Figure 1D. **(C)** KR-2 cells were untreated or treated with UV-inactivated HSV (MOI 5.0) in the presence of cycloheximide (20 µg/mL) for indicated times. Analyses of total levels of IFNAR1 (assessed by IP-IB) and β-actin (as a loading control) are depicted. **(D)** KR-2 cells expressing the indicated Flag-IFNAR1 proteins were treated as shown in panel D. The levels of exogenous IFNAR1 were analyzed by IB using the anti-Flag antibody.

We next tested a possibility that a requirement for prolonged infection and ensuing HSV replication needed to observe the effects of low doses of HSV (MOI 0.1) might be foregone if more viruses are used initially. To this end, we compared the effects of HSV (at MOI 5.0) that was either sham treated or irradiated with UV for inactivation. The latter procedure decreased the titer of this viral preparation from 7×10^7^ to 3 pfu. Remarkably, the treatment of KR-2 cells with a high dose of either active or inactive HSV sufficed for inducing the priming phosphorylation of IFNAR1 within 60–90 minutes (Figure 4A). Furthermore, treatment with either active or inactive HSV comparably decreased the levels of IFNAR1 (Figure 4B) indicating that the downregulation of IFNAR1 can be stimulated by HSV in a manner that does not require virus replication.

The downregulation of IFNAR1 can plausibly occur through diverse mechanisms including an increase in protein degradation and decrease in protein synthesis mediated by translational or pre-translational events (e.g., a decrease in mRNA levels). To determine the role of IFNAR1 proteolytic turnover in this process we used a standard approach of blocking the protein synthesis by treating the cells with cycloheximide. Under these conditions, the treatment of cells with inactivated HSV markedly accelerated the rate of degradation of either endogenous IFNAR1 (Figure 4C) or exogenously expressed Flag-tagged IFNAR1 (Figure 4D). Importantly, inactivated HSV did not increase the rate of proteolytic turnover for the priming site deficient IFNAR1^S532A^ mutant (Figure 4D). Together, these results suggest that HSV rapidly initiates a specific PERK-independent signaling pathway that leads to IFNAR1 priming phosphorylation and degradation.

### Pathogen receptor recognition signaling induces IFNAR1 phosphorylation and degradation

**Figure 5:**
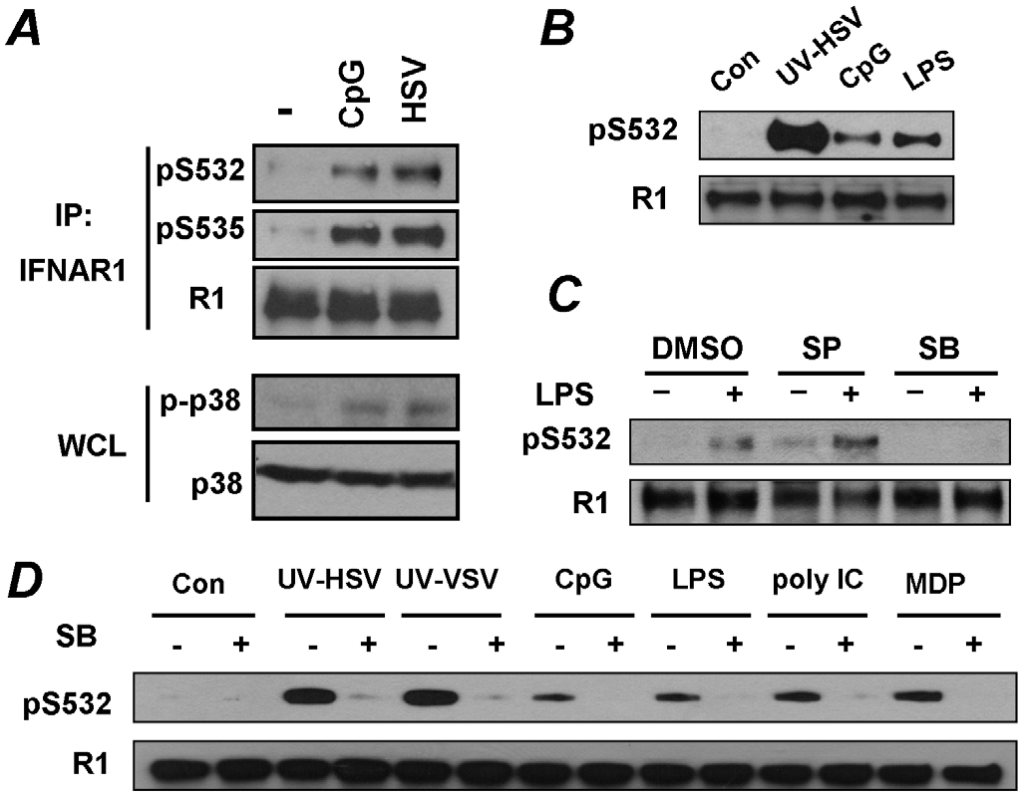
Pathogen recognition receptor signaling induces p38 kinase-dependent priming phosphorylation of IFNAR1. **(A)** KR-2 cells were treated with CpG (10 µM for 30 min) or with HSV (5 MOI for 90 min). The phosphorylation and levels of IFNAR1 were detected after IFNAR1 immunoprecipitation using the indicated antibodies. The levels and activity of p38 kinase in WCL were also analyzed. **(B)** U937 cells were untreated or treated with UV-inactivated HSV (MOI 5), CpG (10 µM) or LPS (10 µg/mL) for 1 h. The phosphorylation and levels of IFNAR1 were analyzed by IP-IB using the indicated antibodies. **(C)** U937 cells were pre-treated with inhibitors of JNK (SP600125, 10 µM) or p38 kinase (SB203580, 10 µM) for 1 h and then treated with LPS (10 µg/ml for 1 h) and analyzed as shown in panel B. **(D)** U937 cells were untreated or pre-treated with the p38 kinase inhibitor SB203580 (10 µM for 1 h) and then treated with the indicated PRR agonists or UV-inactivated HSV (MOI 1), or UV-inactivated VSV (MOI 1) for 1 h and then analyzed as depicted in panel B.

One possibility is that such a signaling pathway could be initiated by the recognition of pathogen patterns within inactivated HSV. HSV was reported to activate Toll like receptors (TLR), including TLR9, via viral genomic DNA [43], [44] as well as TLR2 via an unidentified molecular structure on the virion [45]. While we failed to detect an increase in priming or degron phosphorylation of IFNAR1 in KR-2 cells upon treatment with an activator of TLR2 muramyl dipeptide (MDP, data not shown), such phosphorylation was readily observed when KR-2 cells were treated with HSV or with TLR9 inducer CpG (Figure 5A). Whereas these results do not prove or disprove the participation of specific TLR in IFNAR1 phosphorylation mediated by HSV, they indicate a possibility that the stimulation of PRR signaling in general might lead to the same result.

To test this possibility, we aimed to determine whether other known inducers of PRR signaling were capable of stimulating priming phosphorylation of IFNAR1. To this end, we switched from KR-2 fibrosarcoma cells to the types of cells that actually function to present foreign antigens, and, accordingly, express numerous types of pathogen recognition receptors. The treatment of human monocytic U937 cells with inducers of TLR9 (CpG) or TLR4 (lipopolysaccharide, LPS) led to a robust phosphorylation of IFNAR1 on its priming site (Figures 5B, C). Furthermore, an increase in Ser532 phosphorylation of IFNAR1 in U937 cells was also seen in response to other inducers of PRR such as the TLR3 ligand poly I:C and NOD2/TLR2/TLR4 ligand MDP, and in response to high doses of inactivated VSV (Figure 5D). It is plausible that our previous studies, designed to test the effects of VSV using infection at low MOI and analyzed at the late time point of infection when the induction of UPR is at its maximum, [33] had missed this early effect.

### Role of p38 kinase in pathogen receptor signaling-induced IFNAR1 phosphorylation and degradation

Signaling pathways triggered by the activation of PRR are known to induce a number of important regulatory kinases such as Jun N-terminal kinases (JNK), IκB kinases (IKK), stress-activated p38 protein kinases, and mitogen-activated Erk kinases (reviewed in [6]). The pre-treatment of U937 cells with a pharmacologic inhibitor of p38 kinase (SB203580) prevented an increase in priming phosphorylation of IFNAR1 in response to LPS. Such an effect was not observed when the JNK inhibitor SP600125 was used (Figure 5C). The inhibition of p38 kinase by SB203580 decreased the phosphorylation of S532 in response to all tested inducers of PRR signaling (Figure 5D). These results collectively implicate p38 protein kinase in mediating the priming phosphorylation of IFNAR1 in response to PRR signaling.

**Figure 6:**
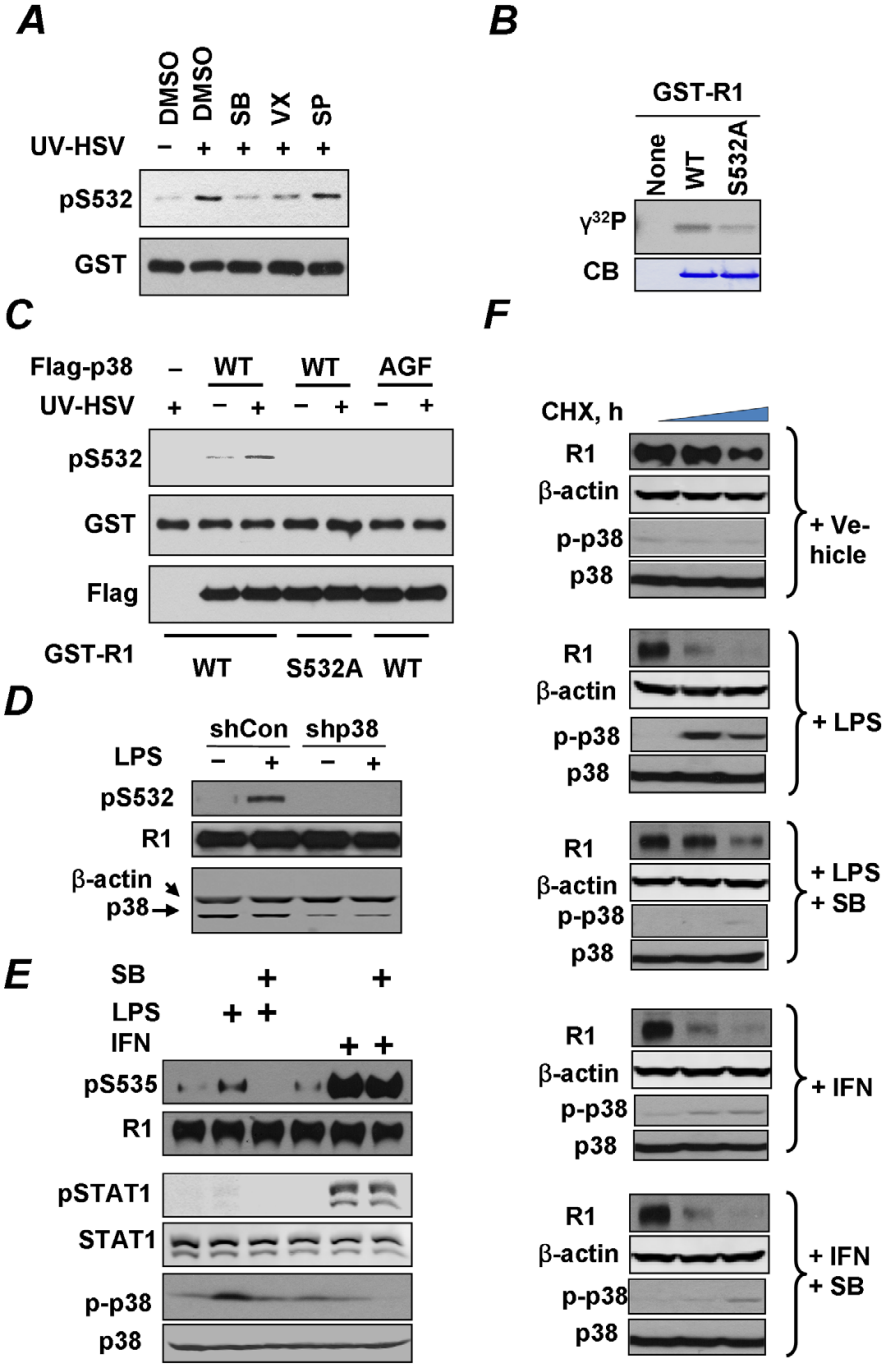
Role of p38 kinase in regulation of IFNAR1 phosphorylation and degradation. **(A)** Lysates from KR-2 cells treated with UV-inactivated HSV (MOI 5 for 90 min) were used as a source of kinase activity in an in vitro assay measuring the phosphorylation of GST-IFNAR1 protein analyzed by IB using anti-pS532 antibody (upper panel) or anti-GST antibody (lower panel). The effects of the p38 kinase inhibitors (SB203580, 50 nM, or VX-702, 20 nM) or the JNK inhibitor (SP600125, 40 nM) are also shown. **(B)** In vitro phosphorylation of GST-IFNAR1 (wild type or S532A mutant) by recombinant active GST-p38α in the presence of γ-^32^P-ATP was determined by conducting a SDS-PAGE followed by an autoradiography. The levels of GST-IFNAR1 were analyzed by Coomassie Blue staining (CB). **(C)** KR-2 cells were transfected with either empty vector (pcDNA3) or Flag-p38α (WT or AGF mutant) as indicated. Cells were either left untreated or treated with UV-inactivated HSV (MOI 5 for 90 min). p38α kinase was immunoprecipitated from the lysates with anti-Flag, and the immunoprecipitates were subject to an *in vitro* kinase assay using GST-IFNAR1 (“GST-R1”, WT, or S532A mutant) as substrates and monitored using the pS532 antibody (top panel). The amounts of GST-IFNAR1 and p38 in the IP reactions are shown. **(D)** U937 cells that received control shRNA or shRNA against p38α kinase were treated with LPS (10 µg/mL for 1 h) and harvested. The phosphorylation and levels of IFNAR1 were analyzed by IP-IB. The levels of p38α kinase in the whole cell lysates are also shown. **(E)** U937 cells were untreated or pre-treated with the p38 kinase inhibitor SB203580 (10 µM for 1 h) and then with either LPS (10 µg/mL for 1 h) or human IFNα (1000 IU/mL for 30 min) as indicated. The phosphorylation of IFNAR1 (by IP-IB) and of p38 kinase and STAT1 (in whole cell lysates by IB) was analyzed. **(F)** Cycloheximide chase analysis of endogenous IFNAR1 in U937 cells treated as in panel E was analyzed using the indicated antibodies. Levels and activation of p38 kinase were also determined.

To further investigate the contribution of the p38 kinase, we used an in vitro assay in which S532 phosphorylation of bacterially-produced GST-IFNAR1 by cell lysates (as a source of kinase activity) was assessed by immunoblotting using a phosho-S532-specific antibody (as in [35]). Under these conditions, lysates from cells treated with UV-inactivated HSV exhibited a greater ability to phosphorylate GST-IFNAR1 on S532 in vitro than lysates from untreated cells. This activity could be tempered by adding p38 inhibitors (SB203580 or VX702) but not by adding the JNK inhibitor SP6000125 (Figure 6A). Furthermore, recombinant p38 kinase was capable of incorporating radiolabeled phosphate groups into the wild type GST-IFNAR1 protein whereas this incorporation was lower when the GST-IFNAR1^S532A^ mutant was used as a substrate (Figure 6B). Finally, the Flag-tagged p38α kinase immunopurified from KR-2 cells was capable of phosphorylating GST-IFNAR1 on S532 in an immunokinase reaction (Figure 6C). This activity was increased when the kinase was purified from cells pre-treated with inactivated HSV. Importantly, no activity was observed when either the catalytically inactive p38^AGF^ mutant was used as a source of kinase or when the phosphorylation-deficient GST-IFNAR1^S532A^ mutant was used as a substrate (Figure 6C). Given that the knock-down of endogenous p38α in U937 cells by shRNA also noticeably decreased the extent of S532 phosphorylation of endogenous IFNAR1 (Figure 6D), these results collectively suggest that the p38 kinase activated by PRR signaling mediates the phosphorylation of the priming site on IFNAR1. Whereas these data indicate that p38 kinase is capable of phosphorylating Ser532, our results do not exclude the possibility that another kinase that associates with p38 and depends on p38 activation could function as a direct priming kinase.

Consistent with the importance of priming phosphorylation in the ligand-independent pathway, the treatment of U937 cells with LPS activated p38 kinase and also stimulated the phosphorylation of S535 within the degron of IFNAR1 (Figure 6E). This phosphorylation was compromised by pre-treating the cells with a p38 kinase inhibitor SB203580. An important observation to note here is that this compound did not affect the Ser535 phosphorylation stimulated by IFNα (Figure 6E). Given that IFNα is a poor inducer of priming phosphorylation and of p38 activation and is capable of stimulating the degron phosphorylation independently of the priming site ([35] and Figure 6E), it appears that the role of p38 kinase in IFNAR1 phosphorylation is largely limited to the ligand-independent pathway. Furthermore, these results (together with a rather poor induction of STAT1 phosphorylation in cells treated with LPS under conditions reported in Figure 6E) render unlikely the possibility that phosphorylation of the IFNAR1 degron in response to PRR signaling is indirectly mediated by autocrine IFNα/β.

We then investigated the effects of PRR signaling on the degradation of IFNAR1. Consistent with previous reports in other cell lines [27], [29], treatment of U937 cells with IFNα markedly increased the rate of IFNAR1 turnover (Figure 6F); a similar effect observed upon the LPS treatment. Importantly, the effects of LPS (but not of IFNα) were blocked by pre-treating the cells with a p38 kinase inhibitor (Figure 6F). These results indicate that PRR signaling specifically accelerates the degradation of IFNAR1 through the activation of p38 kinase whereas this kinase is dispensable for the stimulation of IFNAR1 proteolytic turnover by IFNα.

### Pathogen recognition receptor signaling impedes cellular responses to Type I IFN

**Figure 7:**
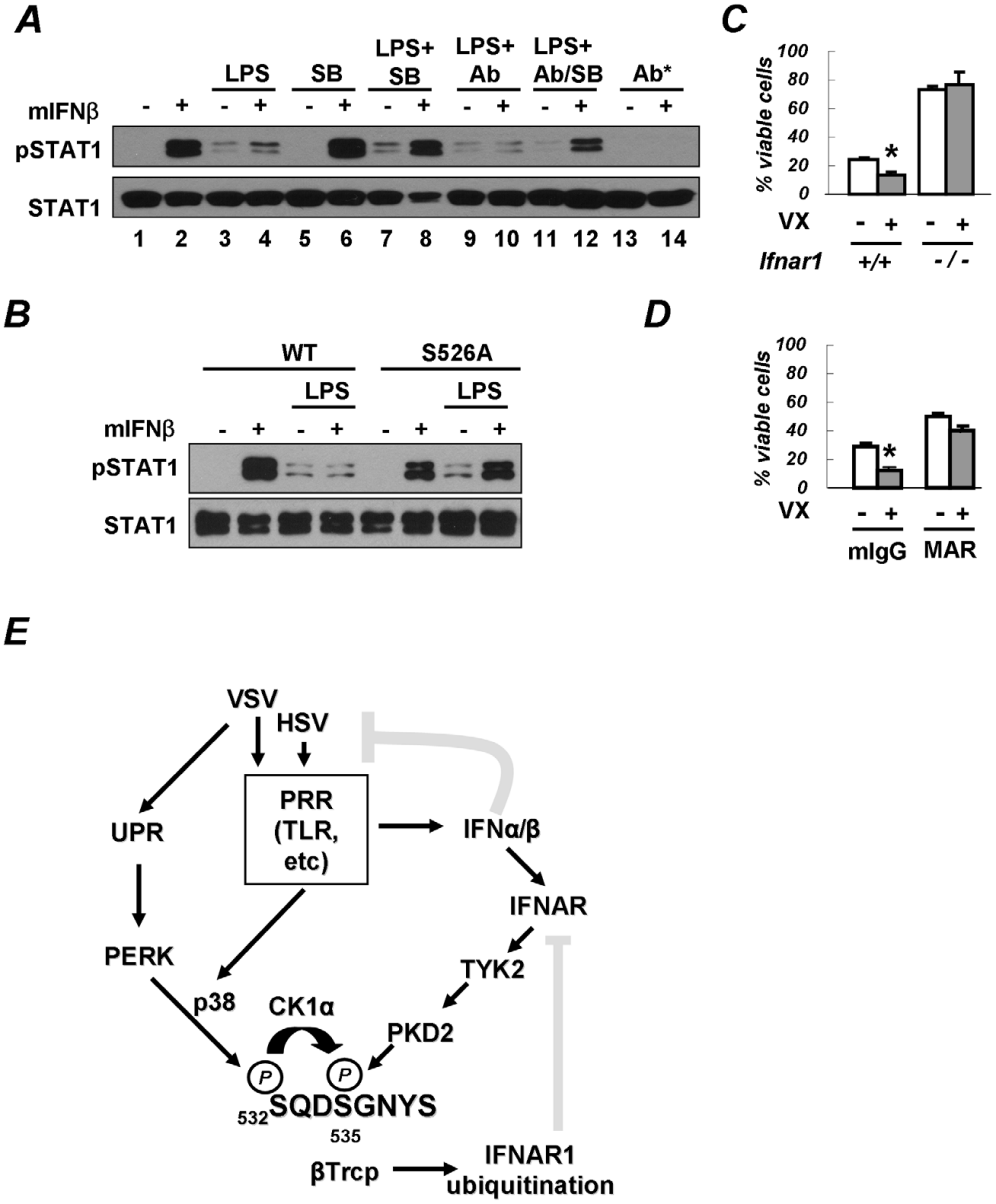
PRR signaling attenuates cellular responses to Type I IFN. **(A)** Mouse bone marrow-derived macrophages (BMM) were pre-treated with LPS (5 µg/mL for 2.5 h) in the presence of either IFNα/β neutralizing antibodies (1000 U/mL) or the p38 kinase inhibitor SB203580 (10 µM) or both, as indicated. Cells were then washed and incubated with murine IFNβ (250 IU/mL for 30 min). The phosphorylation and levels of STAT1 are analyzed in Figure 6E. The asterisk denotes an experiment where neutralizing antibodies were added together with IFNβ. **(B)** BMM from wild type mice or mice expressing the IFNAR1^S526A^ mutant were treated and analyzed as indicated according to the description in panel A. **(C)** Mouse bone marrow-derived dendritic cells (BMDC) from wild type or IFNAR1-null mice were untreated or pre-treated with VX-702 (1 µM for 1 h) and then activated with LPS (2 µg/mL for 48 h). The percent of CD11c-positive and PI-negative viable DCs is depicted as an average of three independent experiments (± S.D.). The asterisk denotes p<0.05 in the *t*-test. **(D)** BMDC from wild type mice were untreated or pre-treated with VX-702 (1 µM for 1 h) and then treated with a neutralizing anti-IFNAR1 antibody (MAR, 10 µg/mL) or normal mouse IgG (mIgG) and activated by LPS (2 µg/mL for 48 h). The percent of CD11c-positive and PI-negative viable DCs is depicted as an average of three independent experiments (± S.D.). The asterisk denotes p<0.05 in the *t*-test. **(E)** Depicted is a hypothetical model that reflects a dual mode of regulation of the IFNα/β responses by PRR signaling (gray blunt arrows represent the inhibitory effects). The ligand-inducible pathway (that involves activation of TYK2 and PKD2) limits the extent of IFNα/β signaling in a cell that has been already exposed to these cytokines. Conversely, the ligand/JAK-independent pathway may (that involves PERK-dependent or independent activation of p38 kinase, priming phosphorylation and ensuing phosphorylation of IFNAR1 degron by CK1α) render a naïve cell less sensitive to its future encounters with Type I IFNs. The activation of PRR both induces production of endogenous Type I IFN and downregulates IFNAR1 in the very same IFN-producing cells. This mechanism may support the viability of the IFN-producing cells and temper an overall activity of IFNα/β pathways.

We next sought to investigate whether the acceleration of IFNAR1 degradation by PRR signaling may attenuate the cellular responses to Type I IFN. We focused on the activation of STAT1 (assessed by its tyrosine phosphorylation) in mouse bone marrow macrophages that robustly responded to exogenous murine IFNβ, and where such a response could be readily abolished if neutralizing antibodies against IFNα and IFNβ were added to the reaction (Figure 7A, lanes 1–2 and 13–14). These settings were then used to examine the effect of PRR activators on the extent of IFN signaling. To this end, we pre-treated cells with various combinations of PRR activator LPS, p38 kinase inhibitor SB203580, and IFNα/β neutralizing antibodies. We then washed off the pre-treatment agents and proceeded to treat the cells with exogenous IFNβ and detect STAT1 phosphorylation.

The pre-treatment of macrophages with LPS alone noticeably increased the basal levels of STAT1 phosphorylation (Figure 7A, lane 3 vs 1). A similar result was observed when pre-treatment with LPS also included anti-IFNα/β neutralizing antibodies (lane 9 vs. 3). This outcome suggests that the effects of LPS on STAT1 phosphorylation should not be attributed to production of endogenous Type I IFN and were likely mediated by other cytokines (for example, IFNλ that is known to be produced upon PRR stimulation [46], [47], [48]). However, the activation of STAT1 in response to the subsequent addition of exogenous IFNβ was noticeably impaired in cells pre-treated with LPS (Figure 7A, compare lanes 2 and 4). This result suggests that the activation of PRR in cells prior to their encounter with Type I IFN may temper future responses of a cell to these cytokines.

Remarkably, the suppressive effect of LPS on IFNβ signaling was partially alleviated when pre-treatment with LPS was carried out in the presence of the p38 kinase inhibitor SB203580 (Figure 7A, lane 8 vs. lane 4). Given that the effects of the p38 inhibitor were largely unaffected by adding the neutralizing antibodies to the pre-treatment combination of LPS and SB203580 (Figure 7A, lane 12 vs. lane 8), it is unlikely that the inhibitor acted through stimulating an additional production of endogenous Type I IFN. Collectively, these results suggest that PRR signaling-induced activation of p38 kinase in cells may attenuate their responses to a subsequent exposure to Type I IFN.

To further determine whether the suppression of IFN signaling by PRR inducers requires IFNAR1 phosphorylation, we used the knock-in mice harboring the IFNAR1^S526A^ mutant, which is insensitive to downregulation in response to HSV infection (Figure 1B). Whereas pre-treatment with LPS dramatically inhibited IFNβ-induced STAT1 phosphorylation in bone marrow macrophages from wild type mice, this inhibition was not seen in cells that express the non-degradable IFNAR1 mutant (Figure 7B). These data collectively suggest that p38 kinase activity-dependent phosphorylation and the downregulation of IFNAR1 in response to PRR signaling decrease the extent of cellular responses to Type I IFN.

Intriguingly, LPS was reported to promote the maturation of human DCs of monocytic origin, a process during which the downregulation of IFNAR1 and the entire Type I IFN receptor had been previously reported [4], [49], [50]. Autocrine/paracrine Type I IFN produced by DCs not only plays an important role in their function but also exerts pro-apoptotic effects on DCs themselves [12], [16]. Given that suppression of cell viability elicited by some of Type I IFN species are most prominent in cells that express high levels of receptor chains [51], it is plausible that IFNAR1 downregulation may help DCs to survive under exposure to their own IFNα/β. To test this hypothesis, we assessed the viability of mouse bone marrow-derived DCs activated by LPS. Treatment with a p38 kinase inhibitor significantly decreased the viability of these cells (Figures 7C). Remarkably, this effect was less evident in DCs that were either derived from IFNAR1-null mice (Figure 7C) or treated with the IFNAR1-neutralizing antibody (Figure 7D) indicating that the activation of p38 kinase may be important for protecting DCs from detrimental effects of Type I IFN. Furthermore, whereas the percent of viable LPS-activated BMDC from the IFNAR1^S526A^ knock-in mice was relatively low (10.7±2.0%), it could be noticeably increased by incubating these cells with the IFNAR1-neutralizing antibody (20.3±3.4%, p<0.01). Similar data were obtained when Annexin V-negative CD11c-expressing cells were analyzed (Figure S2). Taken together, these data suggest that PRR-stimulated p38 kinase-dependent degradation of IFNAR1 results in protection of activated DCs from the detrimental effects of autocrine/paracrine IFNα/β.

## Discussion

We have previously reported that the induction of UPR activates a ligand/JAK-independent signaling pathway that leads to phosphorylation, ubiquitination, and degradation of IFNAR1. This signaling involves stimulation of PERK-dependent priming phosphorylation of IFNAR1 followed by its degron phosphorylation by CK1α. This pathway, which can be activated in response to VSV or HCV infection, plays an important role in regulating the levels of IFNAR1 in naïve cells and in determining the sensitivity of cells to the future exposure to Type I IFN [29], [33], [34], [35].

In the present study, we investigated whether HSV infection also negatively affects IFNAR1 stability and signaling. We found that ligand/TYK2-independent phosphorylation and downregulation of IFNAR1 can indeed be observed in cells infected with HSV (Figure 1). Similar to VSV [35], HSV infection induced the priming phosphorylation of IFNAR1; and this phosphorylation was required for IFNAR1 ubiquitination and downregulation (Figure 2). However, when we next investigated the role of PERK in these processes, the differences between HSV and VSV became apparent. Unlike VSV, HSV infection caused little (if any) increase in the phosphorylation of the major PERK substrate, translational regulator eIF2α (Figure 3A). Although available literature suggests that some of this effect could be attributed to the action of phosphatases directed by the HSV protein γ_1_34.5 [40], our studies together with another report [41] indicated a deficient activation of PERK in cells infected by HSV (Figures 3B–C). Furthermore, genetic experiments using PERK knockdown in human cells and PERK knockout in mouse cells clearly demonstrated that PERK is dispensable for IFNAR1 phosphorylation and downregulation by HSV (Figure 3C–D). Given that high doses of inactivated HSV also stimulated IFNAR1 phosphorylation, downregulation, and degradation (Figure 4), we proposed that there is a novel branch of the ligand-independent pathway. We hypothesized that this signaling branch could be induced by pathogen recognition receptors.

Once this initial hypothesis received support from experiments that used canonical selective activators of PRR signaling (Figure 5), we changed the focus of our study to follow the effects of this signaling. Accordingly, we modified the experimental design and switched to using these canonical activators (due to their commercial availability and better reproducibility over preparations of inactivated virus) and to cell models that specifically reflected the function of pathogenic patterns recognition and ensuing reactions of innate immunity. Our subsequent studies demonstrated that, even in the absence of viral infection, the activation of PRR signaling robustly induces priming phosphorylation and the degradation of IFNAR1 in a manner that requires the activation of p38 kinase (Figures 5–6). Furthermore, p38 kinase-dependent phosphorylation of IFNAR1 leads to IFNAR1 degradation and, accordingly, tempers the responses of cells to their future encounters to IFNα/β (Figures 6–7).

The role of the p38 kinase in these processes is intriguing. On one hand, the results of pharmacologic (Figure 5D) and genetic (Figure 6D) studies implicate p38 kinase in the PRR-induced downregulation of IFNAR1. Furthermore, our biochemical data suggest that p38 kinase is capable of directly phosphorylating the priming site on IFNAR1 in vitro (Figure 4A–C). However, given a known preference of this kinase for proline-directed Ser and Thr residues as phospho-acceptor sites [52] and the fact that the priming site on IFNAR1 does not conform to these criteria, it is plausible that the direct phosphorylation of Ser532 in cells might be carried out by a SB203580-sensitive kinase that associates with p38 kinase and depends on p38 activation.

On another hand, p38 kinase can be also activated by Type I IFN in several types of cells [53], [54], [55]. In fact, within cells that have already encountered it, IFNα/β, p38 kinase activity is proven to contribute to the maximal extent of the IFN-induced transcriptional program [10], [56], [57]. Yet it appears that the ligand-induced phosphorylation and degradation of IFNAR1 does not depend on p38 kinase activity (Figure 6E–F). Indeed, our recent study identified protein kinase D2 as a key TYK2-dependent IFN-inducible kinase that mediates the ligand-stimulated IFNAR1 phosphorylation, ubiquitination, endocytosis and degradation [28]. Furthermore, it appears that the activation of p38 kinase in cells that have not been yet exposed to IFNα/β may temper future sensitivity to these cytokines through an elimination of the receptor (Figure 7E).

Collectively, these studies describe a novel link between an activation of innate immune responses that usually govern production of Type I IFN, with modulation of the extent of cellular responses elicited by these cytokines (Figure 7E). It is plausible that the temporal downregulation of IFNAR1 that precedes or coincides with the peak of IFNα/β synthesis could be important for various aspects of the host defenses. These aspects may include the maintenance of the viability of IFN-producing cells, limiting the extent of IFNα/β pathway, and affecting the sensitivity of the host to the secondary infection.

Among cell types capable of producing IFNα/β, the dendritic cells (DCs) are distinguished with an advanced ability to recognize a variety of pathogenic patterns and, upon this activation, synthesize and secrete Type I IFNs and other cytokines that participate in shaping the immune responses [6], [58]. Activated DCs that produce IFNα/β have to be protected from the detrimental effects of autocrine IFN [4], [12], [16]. Indeed, it has been shown that activated DCs are prone to apoptosis, the extent of which is decreased in cells from IFNAR1-null animals [12], [16]. It has also been demonstrated that, upon their maturation, DCs downregulate Type I IFN receptor [49], [50] though the mechanism underlying this downregulation or its role in DC maturation and survival remain unclear. In this study, we demonstrated that PRR-stimulated p38 kinase-dependent degradation of IFNAR1 leads to an attenuation of Type I IFN signaling and ameliorates its negative effects in DCs (Figure 7). These data suggest that the activation of the signaling pathway that involves phosphorylation and degradation of IFNAR1 may render DCs (and perhaps other IFNα/β-producing cells) refractory to their own Type I IFN.

As indicated by our data (presented in Figure 7), such refractoriness may improve the viability of DCs. In addition, the mechanism described here may plausibly contribute to discontinuing an otherwise potentially harmful cycle of DCs being activated by Type I IFN followed by the production of more of these cytokines and a subsequent greater activation of DCs. Given the well documented role of IFNα/β in the pathogenesis of autoimmune disorders (including systemic lupus erythematosus, psoriasis, and Type 1 diabetes mellitus [17], [18], [19], [20]), temporal downregulation of IFNAR1 might play an important role in protecting the host from such autoimmune reactions.

Whereas the latter outcomes of IFNAR1 degradation stimulated by signaling induced by pathogenic patterns may benefit the host, it could also decrease the ability of some cell types exposed to PRR inducers to mount an appropriate anti-viral response. It remains to be seen whether the suppression of Type I IFN signaling by chronic exposure to other pathogens in other cell types may play a role in the development of secondary viral infections, which have been linked to insufficient IFNα/β function [59]. Future studies in vivo aimed at a further delineation of the mechanisms of IFNAR1 degradation and its role in sensitivity to secondary infections and autoimmune disorders are consequently warranted.

## Materials and Methods

### Plasmids and reagents

Recombinant GST-p38α was purchased (Cell Signaling). Vectors for bacterial expression of GST-IFNAR1 and mammalian expression of human and murine Flag-IFNAR1 were described previously [25], [33], [35]. The plasmids for expression of Flag-p38 (wild type or catalytically inactive AGF) were a generous gift from R. J. Davis. ShRNA constructs for knocking down p38α kinase were from Sigma. Constructs for knock down of PERK were previously described [33]. Recombinant human IFNα2 (Roferon) was from Roche. Recombinant murine IFNβ and mouse IFNα/β neutralizing antibodies were from PBL. LPS, poly IC, MDP and CpG were from InVivogen. Neutralizing antibody against mouse IFNAR1 (MAR) was from Leinco. P38 inhibitor SB203580 and JNK inhibitor SP600125 were from EMD Biosciences. P38 inhibitor VX-702 was from ChemTek. CK1 inhibitor D4476 was from Tocris. All other reagents were from Sigma.

### Viruses

VSV (Indiana serotype, a gift from R. Harty) and HSV-1 (KOS strain, a gift from G. Cohen and R. Eisenberg) were propagated in HeLa cells. These cells were also used to determine the viral titers in serially diluted stocks using methylcellulose method. In experiments requiring inactivated virus, virus suspension was placed in Petri dishes and exposed either to UV-C light (254 nm) for 5 min to achieve the total dose of 1500 J/m^2^ or sham treated for the same time period.

### Cells and gene delivery

Human monocytic U937 and HeLa cells were from ATTC. Replication-deficient lentiviral particles encoding shRNA against GFP (shCON), PERK, p38, or the empty virus control were prepared via co-transfecting 293T cells with three other helper vectors as described previously [33], [35]. Viral supernatants were concentrated by PEG8000 precipitation and were used to infect U937 cells in the presence of polybrene (4 µg/mL, Sigma). Cells were selected and maintained in the presence of 1 µg/mL of puromycin. Human fibrosarcoma 2fTGH-derived IFNAR2-null U5A [39] cells were kindly provided by G. Stark; the isogenic derivatives of TYK2-null 11.1 cells including the KR-2 cells that harbor catalytically inactive TYK2 or WT-5 cells that re-express wild type TYK2 [27] were a generous gift from S. Pellegrini.

### Ethics statement and animals

This study was carried out in strict accordance with the recommendations in the Guide for the Care and Use of Laboratory Animals of the National Institutes of Health. The protocol was approved by the Institutional Animal Care and Use Committee (IACUC) of the University of Pennsylvania (protocol # 800992). Every effort was made to minimize animal suffering. IFNAR1-null mice were kindly provided by D.E. Zhang (UCSD). Bone marrow-derived macrophages (BMM) and bone marrow-derived dendritic cells (BMDC) were produced as described previously [12].

### Immunotechniques and cell viability

These assays were carried out as described previously [34]. Monoclonal antibodies against human IFNAR1 that were used for immunoprecipitation (EA12) or immunoblotting (GB8) were described in detail elsewhere [60]. Antibodies against PERK [33] were kindly provided by J.A. Diehl. Antibodies against p-STAT1, p-p38 (Cell Signaling), phospho-Ser532, phospho-Ser535 (or phospho-Ser523, phospho-Ser526, respectively, in the murine receptor) [26], [35], murine IFNAR1 (LeinCo), STAT1, p38, phospho-PERK (Santa Cruz), Flag, β-actin (Sigma) and ubiquitin (clone FK2, Biomol) were used for immunoprecipitation (IP) and immunoblotting (IB) as described previously [26], [35]. Cell viability assays were analyzed by FACS (BD Calibur) to calculate CD11c positive and propidium iodide negative cell population as described previously [12], [33].

### In vitro kinase assays

Kinase assays were carried out as described previously [29], [34]. In brief, p38 was immunoprecipitated and the immunoprecipitates were incubated with 1 µg of substrates (bacterially-expressed and purified wild type or S532A GST-IFNAR1 mutant) in kinase buffer (25 mM Tris-HCl pH 7.4, 10 mM MgCl_2_, 1 mM NaF, 1 mM NaVO_3_) and ATP (1 mM) at 30°C for 30 min. Samples were then separated by 10% SDS-PAGE and analyzed by immunoblotting with phospho-specific antibodies.

## Supporting Information

Figure S1 PCR analysis of DNA from tails of back-crossed chimeric Ifnar1S526A mice of the indicated gender. Presence of the SA alleles in 3 founders is indicated by a PCR product that migrates slower due to a remaining loxP site.
(TIF)

Figure S2 BMDC from wild type mice or IFNAR1 S526A mutant mice were untreated or treated with neutralizing anti-IFNAR1 antibody (MAR, 10 µg/mL) or normal mouse IgG (mIgG) and activated by LPS (2 µg/mL for 48 h). Percent of CD11c-positive and Annexin V-negative viable DCs is depicted as an average of three independent experiments (±S.D.).
(TIF)
