# Supplementary figures and images for: Pathogen Recognition Receptor Signaling Accelerates Phosphorylation-Dependent Degradation of IFNAR1
Source: PLoS Pathog. 2011 Jun 9;7(6):e1002065. doi: 10.1371/journal.ppat.1002065 (PMC3111542; doi:10.1371/journal.ppat.1002065)

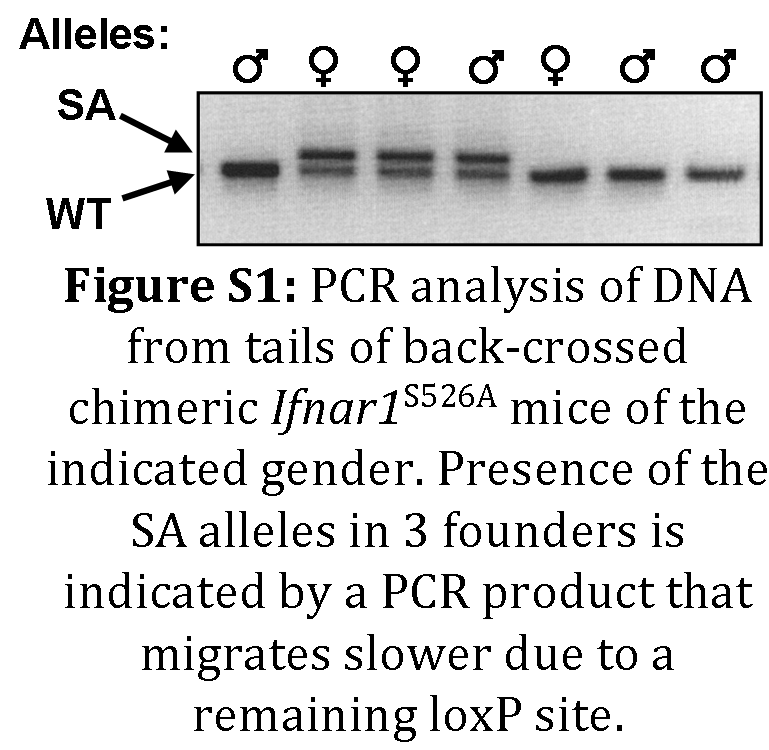

Supplement: Figure S1 — PCR analysis of DNA from tails of back-crossed chimeric Ifnar1S526A mice of the indicated gender. Presence of the SA alleles in 3 founders is indicated by a PCR product that migrates slower due to a remaining loxP site. (TIF) [file ppat.1002065.s001.tif]

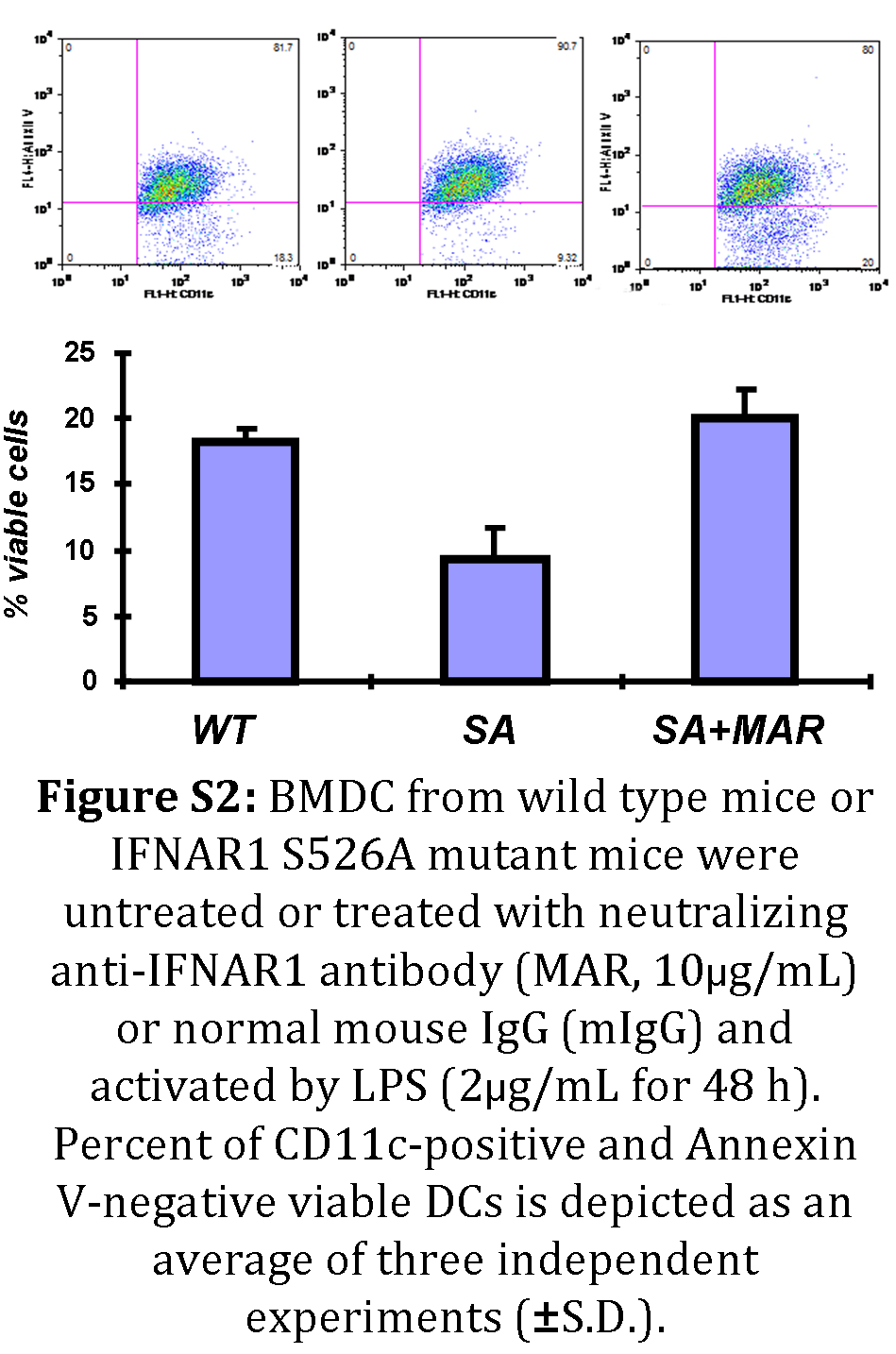

Supplement: Figure S2 — BMDC from wild type mice or IFNAR1 S526A mutant mice were untreated or treated with neutralizing anti-IFNAR1 antibody (MAR, 10 µg/mL) or normal mouse IgG (mIgG) and activated by LPS (2 µg/mL for 48 h). Percent of CD11c-positive and Annexin V-negative viable DCs is depicted as an average of three independent experiments (±S.D.). (TIF) [file ppat.1002065.s002.tif]
